# Frozen Elephant Trunk: A technique which can be offered in complex pathology to fix the whole aorta in one setting

**DOI:** 10.1186/1749-8090-6-66

**Published:** 2011-05-08

**Authors:** John Kokotsakis, Vania Anagnostakou, Theodoros Kratimenos, Hutan Ashrafian, Thanos Athanasiou

**Affiliations:** 1 Cardiac Surgery Department, Evangelismos General Hospital, Athens, Greece; 2 Radiology Department, Evangelismos General Hospital, Athens, Greece; 3 Department of Cardiothoracic Surgery, Imperial College Healthcare NHS Trust and Department of Surgery and Cancer, Imperial College London, London, UK

**Keywords:** Aorta, Frozen Elephant Trunk, Dissection, Aneurysm

## Abstract

We report a case of treating complex aortic pathology with the use of the Frozen Elephant Trunk technique in a patient with chronic type B aortic dissecting aneurysm associated with arch and ascending aorta dilatation, proximal aortic disease and coronary disease. The case was further complicated due to the involvement of the abdominal vessels and preexisting femoral to femoral crossover bypass. In addition the patient had a tracheostomy for laryngeal cancer.

We emphasize the role of the Frozen Elephant Trunk to fix the whole aorta in one setting with special attention given to the changes taking place in vascular perfusion following correction and reconstitution of the true lumen.

## Background

The optimal surgical management of chronic type B dissecting aneurysms concomitant with proximal aortic and cardiac pathology is controversial [1]. A conventional single-stage procedure with combined incisions (thoraco-sternotomy or bilateral anterior thoracotomy) is associated with increased morbidity. A staged procedure may be used, but death due to rupture of the remaining aneurysmal aorta during the interval between the first and second stages of the procedure have been recorded [2]. Arch debranching with stent grafting seems to require a similar arch exposure with compromised durability. We report the surgical treatment of a patient with complex pathology, in one stage, using the frozen elephant trunk (FET) procedure.

## Case Presentation

A 65-year-old man with hypertension was admitted to our hospital because of back pain due to a large 9 cm aneurysm of the proximal descending aorta originating from a chronic type B dissection.

The patient had suffered a complicated type B dissection 18 months earlier and had undergone a femoral-femoral bypass for left leg ischaemia and temporary haemodialysis for renal failure due to right kidney hypoperfusion. He had also undergone a total laryngectomy with permanent tracheostomy for laryngeal cancer one year previously.

**Figure 1:**
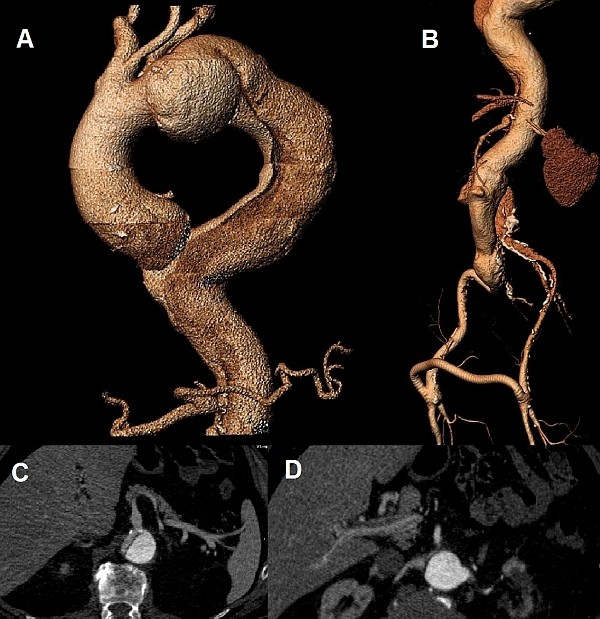
**Pre-operative images of the aorta**. (a.) 3D CT reconstruction demonstrating the dissecting type B aneurysm and the dilatation of the arch and ascending aorta (b.) 3D CT reconstruction image of the dissected abdominal aorta and the patent femoro-femoral bypass graft (c.) Axial CT image showing the origin of the celiac trunk from the true lumen (d.) Axial CT image demonstrating the origin of the left renal artery from the false lumen and right renal artery from the true lumen.

Preoperative evaluation included a coronary angiogram that revealed 90% stenosis of the proximal Left Anterior Descending (LAD) artery and total occlusion of the Right Coronary Artery (RCA). Transthoracic echocardiographic analysis revealed moderate (2+/4+) aortic regurgitation with left ventricular ejection fraction of 50%. Carotid duplex ultrasound scan demonstrated mild (<50%) carotid artery stenosis. Computed tomographic angiography (CTA) (Figure 1a and 1b) identified a degenerative aneurysm of the ascending aorta (5 cm) and aortic arch (5 cm) which had a bovine configuration (common origin of the innominate and left common carotid artery). There was also post-dissection type B aneurysm of the descending aorta (9 cm) starting just below the left subclavian artery and tapering to the celiac trunk. There was severe compression of the true lumen from the false lumen, the celiac and right renal artery originated from the true lumen (Figure 1b and 1c, superior mesenteric artery from both lumens, left renal artery from the false lumen (Figure 1d). The right femoral artery was perfused from the false lumen while the left from the compressed true lumen and the functioning femoral-femoral bypass.

CTA was used to calculate the appropriate hybrid stent-graft size. Blood tests revealed chronic renal insufficiency (Cr ≥ 2.0 mg/dl). Oncologic evaluation was negative for recurrent or metastatic disease from previous laryngeal cancer.

## Surgical Technique

Anaesthetic induction was achieved with standard technique including administration of sodium pentothal, sevofluorane, fentanyl and muscle relaxant. Invasive monitoring included the use of right radial and left radial arterial lines, a pulmonary artery catheter and a foley catheter with temperature probe to measure bladder temperature as an indicator of core body temperature. Cerebral monitoring was achieved by means of transcutaneous cerebral oximetry (INVOS 3100-SD; Troy, Mich) and electroencephalogram. Transesophageal echocardiography (TEE) was also performed. After systemic heparinization and before incision, a guide-wire was inserted through the left femoral artery in the true lumen of the descending thoracic aorta under fluoroscopic and TEE control. A catheter for cerebrospinal fluid (CSF) drainage was also inserted.

A median sternotomy was carefully performed leaving enough skin tissue in the suprasternal area in order to avoid the low-lying permanent tracheostomy. A right subclavicular incision was also made and the right axillary artery was exposed. Cardiopulmonary bypass (CPB) was instituted with an arterial cannula introduced into the right axillary artery through an interposed 8 mm Dacron graft and with a venous single two-stage cannula introduced into the right atrium. The arterial line of CPB circuit was bifurcated, one arm for axillary artery perfusion and the other arm for later perfusion of the side branch of the arch graft. CPB was commenced and flow was maintained between 2.2-2.4 L per min per square meter of body surface area. A retrograde cardioplegia catheter was placed in the coronary sinus via the right atrium. Left ventricular decompression was achieved with a vent placed through the right superior pulmonary vein. Active cooling was started to a bladder temperature of 25°C. Upon cardiac fibrillation, a cross-clamp was placed across the ascending aorta and resected above the coronary ostia in the sinotubular junction. Myocardial arrest was achieved with cold crystalloid cardioplegia 25 ml kg^-1 ^(Custodiol, Koehler CHEMIE, Alsbach-Haenlein, Germany) delivered both retrograde and antegrade through the left coronary ostium.

A CABG × 2 was first performed with saphenous vein grafts to the RCA and LAD. An additional dose of 400 ml cardioplegia was administered through the graft of the RCA to augment myocardial protection of the right ventricle. Aortic valve replacement followed using a mechanical 22 mm Overline Sorin supra-annular aortic prosthesis.

Once the target bladder temperature of 25°C was reached the CPB flow was reduced to one liter per min and the common trunk (CT) of the innominate and left common carotid artery was clamped in order to obtain selective bilateral antegrade cerebral perfusion. The common trunk and LSA were transected 1 cm distal from their origin. The proximal stump of LSA in the arch was ligated.

The stent-graft system (28 mm E-vita open plus; Jotec Inc., Hechingen, Germany) was then introduced in an antegrade manner in the true lumen of the descending aorta over a stiff guide-wire and released with a pull back system. The incorporated Dacron graft was pulled back, cut to a minimum and sutured to the transected distal arch with interrupted horizontal mattress 3-0 polypropylene sutures with external Teflon strip reinforcement. A 14 mm Hegar dilator was inserted inside the E-vita prosthesis in order to check the opening of the stent-graft.

A Dacron vascular prosthesis with one side branch (28 × 10 mm) was then prepared and anastomosed with the cuff composed by the native aorta and the E-vita prosthesis. Systemic perfusion was antegradely restored through the side branch of the graft. The LSA was implanted to the arch prosthesis through an interposed 8 mm Dacron graft and released to circulation. The common trunk of the innominate and left common carotid artery was directly reimplanted to an opening of the arch graft and after complete de-airing systemic circulation to the brain was restarted from the side branch of the arch graft, while selective antegrade cerebral perfusion (SACP) from the right axillary artery was stopped. The ascending aorta was replaced with another Dacron (30 mm) prosthesis attached proximally to the sinotubular junction and distally to the arch prosthesis. The proximal anastomoses of the saphenous vein grafts were created in the ascending aorta graft.

The synthetic grafts were covered with a strip of human pericardium excluding them from the sternal wound. The CPB time was 340 min, SACP time 95 min, lower body circulatory arrest time 52 min, myocardial ischaemic time 290 min. Deployment of the stented end of the hybrid prosthesis required 12 min. The correct opening of the stent-graft was controlled with TEE.

**Figure 2:**
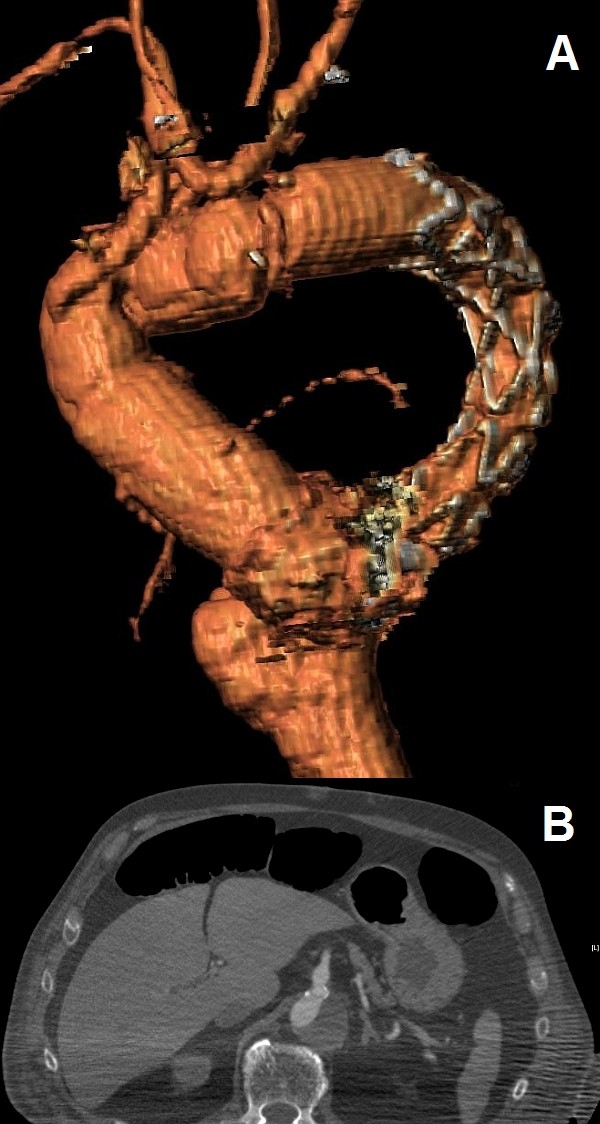
**Postoperative images of the aorta **. (a.) 3D CT reconstruction image of the thoracic aorta showing complete replacement of ascending aorta and aortic arch, the FET in the descending thoracic aorta and the saphenous vein grafts originating from the ascending aorta. (b.) Axial CT image demonstrating good flow in the celiac trunk.

The patient stayed in the intensive care unit for 6 days. He was extubated 18 h after the operation and remained haemodynamically stable and neurologically intact. However he experienced deterioration of his renal function and required temporary haemodialysis for three weeks. A CTA scan was performed at 3 months after surgery and revealed complete thrombosis of the aneurysmal false lumen and expansion of the true lumen of the descending thoracic aorta (Figure 2a). The visceral arteries were perfused from the true lumen (Figure 2b and 2c) while flow in the pre-existing femoral-femoral bypass was reversed (left to right).

## Conclusions

Complex thoracic aortic disease involving the ascending aorta, the aortic arch and the descending aorta still represents a challenge for the cardiothoracic surgeon. It requires either a two-stage approach employing the elephant trunk procedure or an extensive single stage operation performed through a clamshell incision. Both approaches have significant disadvantages that lead to the development by some surgeons, of the one-stage hybrid stent-graft procedure [3]. This new approach, also termed the FET technique, consists of treating the combined lesions of the thoracic aorta during a single-stage procedure combining endovascular treatment with conventional surgery.

In our patient we utilized the commercially available E-Vita open hybrid prosthesis which has a stented distal segment implanted into the dissected descending aorta through the opened aortic arch, the FET, while the proximal non-stented segment was used for conventional replacement of the upstream aorta. The procedure was performed through a median sternotomy, thereby fascilitating the additional surgery needed on the heart and the ascending aorta.

Debranching of the supra-aortic vessels, followed by immediate antegrade or retrograde stent-grafting of the aortic arch and descending aorta was unsuitable for our patient due to aneurysmal dilatation of the entire thoracic aorta and concomitant cardiac pathology.

Experience with the FET technique is limited but early results seem to be encouraging [4]. Our limited experience confirms this trend [5]. However these are very complex and time-consuming operations and good results can be obtained only if good strategies of myocardial, cerebral and visceral protection are adopted. Myocardial protection can be achieved with the administration of custodial cardioplegia as a single dose of 20-25 ml per kg, and guarantees 3 h of myocardial ischaemia. In exceptional cases of myocardial ischaemia larger than 3 h, a half dose of cardioplegia can be repeated.

Selective antegrade cerebral perfusion is the method of choice for brain protection during aortic arch surgery and in these challenging and time-consuming operations, as in our case, its use is essential. An increased risk of spinal cord injury (SCI) after FET has been reported by some authors, although the exact mechanism underlying spinal cord ischaemia is not fully understood. Risk factors include occlusion of vital intercostal arteries, perioperative hypotension and prior abdominal aortic aneurysm repair [6]. The methods that we applied to avoid SCI included CSF drainage, limited deployment of stent at the T7 level or higher, early perfusion of the distal thoracic aorta (after completion of the distal anastomosis) through the side-branch of the arch graft and early reimplantation and reperfusion of the LSA.

The current indications for the FET procedure are extensive aneurysms involving the three levels of the thoracic aorta (ascending, arch and descending), chronic dissection after acute type A aortic dissection repair or type B dissection associated with ascending or arch aneurysms. The application of this technique in acute dissections is still controversial. The practical problems with routine use of this procedure in chronic dissections are the necessity to identify true and false lumens and to know if there are visceral vessels arising from the false lumen. Visceral ischaemia after the complete sealing of the false lumen could occur if the abdominal arteries arise from the false lumen itself and no re-entry is present in the distal aorta. This is a rare possibility because there is always re-entry in the descending, abdominal aorta through the iliac arteries. However we believe that the FET procedure should be contraindicated if re-entry sites are not visualized in the distal descending and/or abdominal aorta and the visceral arteries arise from the false lumen. In our patient there was no visceral ischaemia postoperatively although we found hypoperfusion of the left kidney, as the left renal artery was arising from the false lumen, and reversal of flow in the pre-existing femora-femoral bypass.

The correct positioning of the E-vita prosthesis can be achieved using a guide-wire positioned in the true lumen under fluoroscopic guidance and TEE before starting the operation. Moreover, TEE provides useful information about the correct opening of the stent-graft.

In conclusion, the FET represents a new surgical paradigm for the effective management of complex thoracic aortic disease. However strict postoperative monitoring of these patients is required in order to detect the possible evolution of the aortic lesions, which can demand prompt intervention. The future management of these patients can also be enhanced with further long-term follow-up data to support clinical decisions and interventional strategies.

## Consent

Written informed consent was obtained from the patient for publication of this Case report and any accompanying images. A copy of the written consent is available for review by the Editor-in-Chief of this journal.

## Competing interests

The authors declare that they have no competing interests.

## Authors' contributions

All authors participated in the coordination and drafting of this text. All authors read and approved the final manuscript.
